# The Emerging Role of m6A Modification in Regulating the Immune System and Autoimmune Diseases

**DOI:** 10.3389/fcell.2021.755691

**Published:** 2021-11-16

**Authors:** Yimeng Wang, Lifang Li, Jiaqi Li, Bin Zhao, Gan Huang, Xia Li, Zhiguo Xie, Zhiguang Zhou

**Affiliations:** ^1^ National Clinical Research Center for Metabolic Diseases, Key Laboratory of Diabetes Immunology (Central South University), Ministry of Education, and Department of Metabolism and Endocrinology, The Second Xiangya Hospital of Central South University, Changsha, China; ^2^ Department of Ultrasound, The Third Xiangya Hospital of Central South University, Changsha, China

**Keywords:** RNA modifications, N6-methyladenosine, autoimmune disorders, innate immunity, adaptive immunity

## Abstract

Over the past several decades, RNA modifications have rapidly emerged as an indispensable topic in epitranscriptomics. N6-methyladenosine (m6A), namely, methylation at the sixth position of an adenine base in an RNA molecule, is the most prevalent RNA modification in both coding and noncoding RNAs. m6A has emerged as a crucial posttranscriptional regulator involved in both physiological and pathological processes. Based on accumulating evidence, m6A participates in the pathogenesis of immune-related diseases by regulating both innate and adaptive immune cells through various mechanisms. Autoimmune diseases are caused by a self-destructive immune response in the setting of genetic and environmental factors, and recent studies have discovered that m6A may play an essential role in the development of autoimmune diseases. In this review, we focus on the important role of m6A modification in biological functions and highlight its contributions to immune cells and the development of autoimmune diseases, thereby providing promising epitranscriptomic targets for preventing and treating autoimmune disorders.

## Introduction

Epigenetics, a link between genetic factors and environmental factors, refers to heritable modifications that regulate gene expression in the absence of nucleotide sequence alterations. Classical epigenetic mechanisms comprise DNA modifications, histone modifications and noncoding RNAs (ncRNAs). Over the past several decades, RNA modifications have emerged as new epitranscriptomic modifications, enriching the regulatory mechanisms of gene expression and providing novel insights into and strategies for exploring the underlying pathogenesis of diseases. N6-methyladenosine (m6A), the most abundant and widespread RNA modification, has been identified in coding RNAs (messenger RNAs, mRNAs) and ncRNAs, including transfer RNAs (tRNAs), ribosomal RNAs (rRNAs), microRNAs (miRNAs), small nuclear RNAs (snRNAs), long noncoding RNAs (lncRNAs) and circular RNAs (circRNAs) (Niu et al., 2013; Chen et al., 2019; Lence et al., 2019; Li et al., 2020a). m6A is highly conserved and is installed predominantly in specific regions near stop codons, in internal long exons and in 3′ untranslated regions (3′UTRs) (Dominissini et al., 2012; Meyer et al., 2012; Wang et al., 2020a). More specifically, m6A is preferentially installed at the consensus motif RR-m6A-CH (R = G/A; H = A/C/U)(Batista, 2017).

**FIGURE 1:**
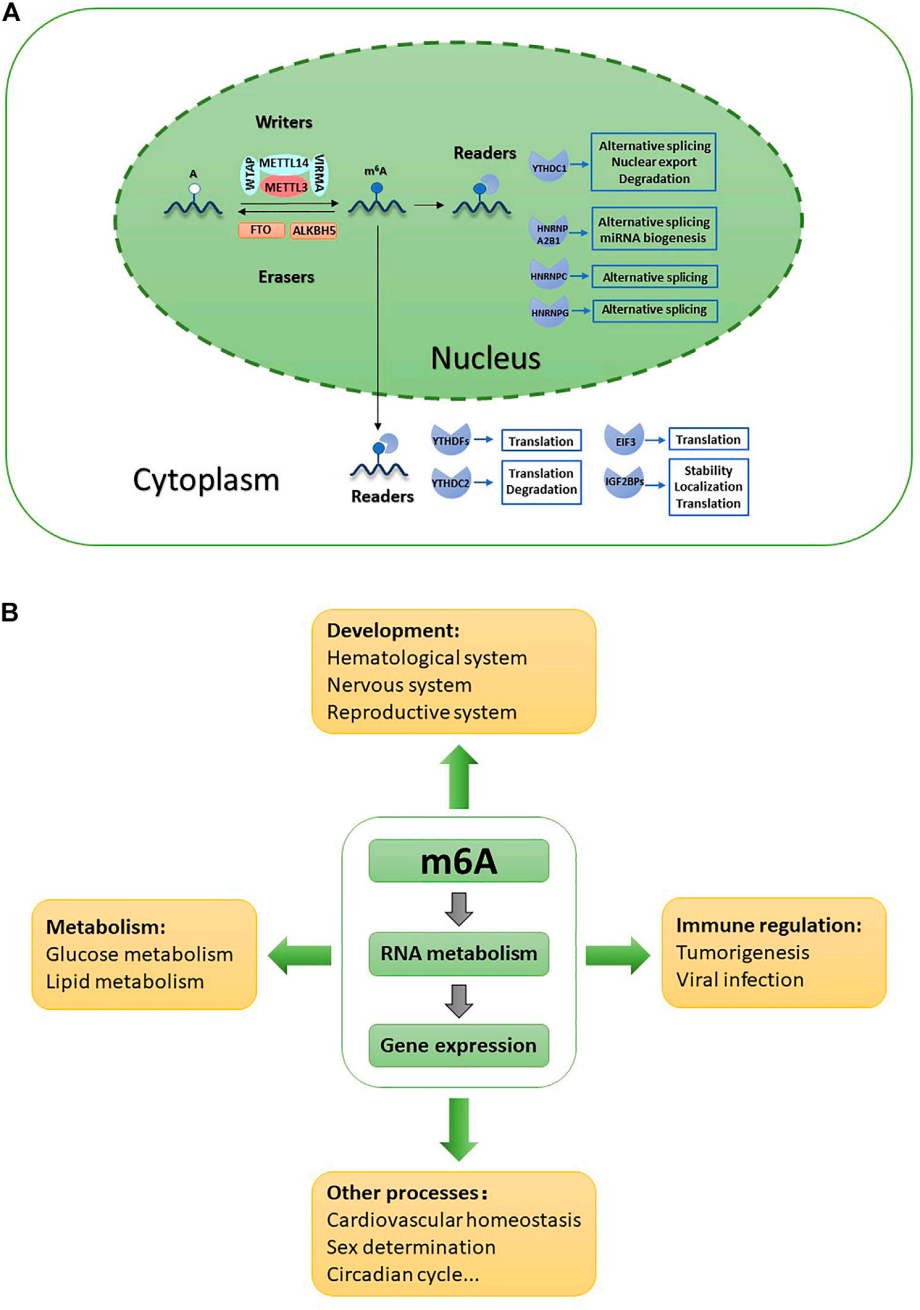
Role of m6A in various biological functions. **(A)** m6A is installed by writers, removed by erasers and recognized by nuclear readers and cystoplasmic readers. m6A is involved in all aspects of RNAs metabolism and activity. **(B)** m6A is involved in both physiological and pathological processes through modulating gene expression.

The regulatory proteins involved in m6A modification fall into three categories: “writers,” “erasers” and “readers” (Table 1; Figure 1A). m6A writers are methyltransferase complexes containing multiple subunits that install m6A cotranscriptionally at specific sites in target mRNAs. Methyltransferase-like 3 (METTL3), the only active catalytic component of the writer complex, exhibits catalytic activity independently and functions synergistically with METTL14 by forming a stable heterodimer. Many auxiliary subunits ensure efficient installation of m6A modification and determine the specificity of writers, including Wilms tumor 1-associated protein (WTAP), Vir-like m6A methyltransferase associated (VIRMA), and RNA binding motif protein 15/15B (RBM15/15B) (Shi et al., 2019). Erasers are RNA demethylases that remove the methyl group from m6A. The discovery of RNA demethylases suggested that m6A modification may be a reversible and dynamic process. Only two natural RNA demethylases have been identified to date, namely, fat mass and obesity-associated protein (FTO) and ALKB homolog 5 (ALKBH5), both of which belong to the ALKB family of proteins (Jia et al., 2011; Zheng et al., 2013). Recently, flavin mononucleotide (FMN) was identified as a novel artificial molecular demethylase (Xie et al., 2019). Readers are RNA binding proteins that mediate the fate of target transcripts and regulate downstream biological functions by preferentially recognizing and binding to modified sites (Batista, 2017; He et al., 2019; Zhang et al., 2019). Readers are classified as direct readers or indirect readers according to their interaction patterns with RNAs. Direct readers, such as YTH family members, selectively and directly bind to m6A sites, while indirect readers, such as heterogeneous nuclear ribonucleoprotein G (HNRNPG), indirectly bind to m6A sites based on the ‘‘m6A switch” mechanism (Dai et al., 2018; Zaccara et al., 2019).

**TABLE 1:** Main functions of the three major groups of m6A-related proteins.

Category	Proteins	Main functions	References
Writers	METTL3	Catalyzes m6A formation	Tong et al. (2018a); Zhang et al. (2019)
	METTL14	Promotes the catalytic activity of METTL3	Wang et al. (2016); Batista (2017); Tong et al. (2018a); Zaccara et al. (2019); Zhang et al. (2019)
	WTAP	Ensures proper localization of the METTL3-METTL14 complex	Ping et al. (2014); Zhang et al. (2019)
		Recruits target RNAs and other functional factors	Zhang et al. (2019)
	VIRMA	Mediates m6A deposition at specific regions of target mRNAs	Yue et al. (2018)
	RBM15/15B	Recruits the writer complex to specific regions	Patil et al. (2016)
		Modulates m6A deposition on XIST	Patil et al. (2016)
	METTL16	Installs m6A on snRNAs and mRNAs to regulate SAM homeostasis	Pendleton et al. (2017)
	CBLL1	Interacts with WTAP	Růžička et al. (2017)
	ZC3H13	Facilitates the nuclear localization of the writer complex	Knuckles et al. (2018); Wen et al. (2018)
	ZCCHC4	Installs a single m6A modification on rRNA	Pinto et al. (2020)
		Modulates mRNA translation	Ma et al. (2019)
Erasers	FTO	Removes m6A	Jia et al. (2011)
		Removes m6Am	Mauer et al. (2019)
		Demethylates other sites in certain snRNAs and specific tRNAs	Wang et al. (2014); Mauer et al. (2019)
	ALKBH5	Removes m6A	Zheng et al. (2013)
		Targets ncRNAs separately from mRNAs	Wang et al. (2014)
		Participates in germ cell development	Zheng et al. (2013)
		Modulates RNA metabolism and nuclear RNA export	Zheng et al. (2013); Zhang et al. (2017a)
	FMN	A newly identified artificial molecular demethylase	Xie et al. (2019)
Readers	YTHDF1	Promotes the efficient translation of target mRNAs	Meyer et al. (2015); Wang et al. (2015)
	YTHDF2	Accelerates RNA degradation and inhibits the translation of mRNAs	Meyer et al. (2015); Wang et al. (2015)
	YTHDF3	Promotes RNA translation with YTHDF1	Shi et al. (2017)
		Inhibits the translation of mRNAs with YTHDF2	Shi et al. (2017)
	YTHDC1	Modulates alternative RNA splicing	Xiao et al. (2016)
		Regulates nuclear export	Xiao et al. (2016)
		Accelerates the decay of transcripts to maintain SAM levels	Shima et al. (2017)
		Suppresses gene expression involving X chromosome inactivation	Patil et al. (2016)
	YTHDC2	Promotes the efficient translation of its target transcripts	Kretschmer et al. (2018)
		Mediates the subsequent degradation of its target transcripts	Kretschmer et al. (2018)
	IGF2BPs	Regulates the stability, localization and translation of target RNAs	Degrauwe et al. (2016); Huang et al. (2018)
	EIF3	Initiates and promotes cap-independent translation	Meyer et al. (2015)
	HNRNPA2B1	Modulates alternative splicing	Alarcón et al. (2015a)
		Promotes mature miRNA biogenesis	Alarcón et al. (2015a); Coker et al. (2019)
	HNRNPC	Involved in premRNA processing, including splicing	Liu et al. (2015)
	HNRNPG	Involved in premRNA processing, including splicing	Liu et al. (2017)

ALKBH5, ALKB homolog 5; CBLL1, Casitas B-lineage lymphoma-transforming sequence-like protein 1; FMN, flavin mononucleotide; FTO, fat mass and obesity-associated protein; HNRNPA2B1/C/G, heterogeneous nuclear ribonucleoprotein A2B1/C/G; METTL3/14/16, methyltransferase-like 3/14/16; IGF2BPs, insulin-like growth factor 2 binding proteins; RBM15/15B, RNA binding motif protein 15/15B; VIRMA, Vir-like m6A methyltransferase associated; WTAP, Wilms tumor 1-associated protein; YTHDF1/2/3, YTH domain-containing family 1/2/3, YTHDC1/2, YTH domain-containing 1/2; ZC3H13, zinc finger CCCH domain-containing protein 13; ZCCHC4, zinc finger CCHC-type containing 4.

Autoimmune diseases result from a self-destructive immune response initiated by an impaired immune tolerance mechanism. This group of diseases imposes a substantial burden on health services, economic development and quality of life due to their slow progression, the difficulty in diagnosis because of their heterogeneous clinical manifestations and the numerous side effects occurring during immunosuppressive therapy (Anaya, 2012; Wardowska, 2021). However, the precise cellular and molecular mechanisms underlying autoimmune diseases have remained poorly understood until recently. Epitranscriptomic mechanisms have been widely recognized to play fundamental roles in the pathogenesis of immune-related diseases (Wu et al., 2017; Li et al., 2021a). Both innate and adaptive immunity clearly participate in the occurrence and progression of autoimmune diseases (Bluestone and Bour-Jordan, 2012; Wahren-Herlenius and Dörner, 2013). Numerous studies have recently characterized an essential role for m6A in many aspects of the immune system, including cell development, differentiation, activation, migration and function, indicating that m6A may contribute to the pathogenesis of autoimmune disorders.

In this review, we summarize the crucial role of the m6A modification in regulating cellular biological functions and highlight its contributions to the immune system and the development of autoimmune diseases, thereby providing novel insights into the pathogenesis of autoimmune disorders and potential targets for epitranscriptomic therapy.

## Roles of m6A in Various Biological Functions

m6A modification is precisely regulated by writers, erasers and readers and is involved in all aspects of RNA metabolism; moreover, its effects are not limited to mRNAs (Figure 1A). Through regulating gene expression, m6A is involved in diverse biological process including development, metabolism, immunity regulation, sex determination, circadian rhythms, and cardiovascular system homeostasis (Hu et al., 2020; Gu et al., 2021) (Figure 1B).

### Roles of m6A in RNA Metabolism and Activity

m6A and related proteins regulate almost all aspects of RNA metabolism and activity, thus modulating gene expression under physiological and pathological conditions. On the one hand, m6A is involved in the processing, alternative splicing, nuclear export, translation and degradation of mRNAs. FTO cooperates with METTL3 to regulate poly(A) sites and change the length of the 3′UTR (Bartosovic et al., 2017). In addition, FTO regulates alternative mRNA splicing not only by inhibiting the binding ability of serine and arginine-rich splicing factor (SRSF) 2 protein in an m6A-dependent manner but also by targeting m6Am during the biogenesis of snRNAs, which are integral spliceosome components and are involved in regulating premRNA splicing (Zhao et al., 2014; Mauer et al., 2019). YTH domain-containing 1 (YTHDC1) regulates alternative splicing by recruiting SRSF3 to promote exon inclusion and facilitates the binding of methylated mRNAs to nuclear export factor 1 (NXF1) to modulate their nuclear export (Xiao et al., 2016). YTH domain-containing family 1 (YTHDF1) promotes the efficient translation of target mRNAs in a cap-independent manner, particularly through its interaction with eukaryotic initiation factor 3 (eIF3), while YTHDF2 accelerates RNA degradation and inhibits protein translation by preferentially binding m6A in the 3’ UTR and then recruiting the CCR4-NOT complex (Meyer et al., 2015; Wang et al., 2015; Du et al., 2016).

On the other hand, m6A is involved in regulating ncRNA metabolism and activity, including miRNA biogenesis, circRNA translation, and lncRNA stability and localization. With the assistance of METTL3 methylation activity, HNRNPA2B1 promotes mature miRNA biogenesis by recruiting DiGeorge syndrome chromosomal region (DGCR) 8 to primary miRNAs(Alarcón et al., 2015a; Alarcón et al., 2015b). METTL14 promotes the processing of pri-miR126 by directly recruiting DCGR8 (Ma et al., 2017). METTL3 has been reported to indirectly regulate miRNA expression and facilitate the translation initiation of circRNAs through an m6A-dependent mechanism (Karthiya and Khandelia, 2020). RBM15/15B alters the deposition of m6A on X-inactive specific transcript (XIST) by promoting the methylation of XIST, resulting in X-chromosome inactivation and gene silencing (Patil et al., 2016). METTL3 overexpression significantly increases the localization of the lncRNA RP11 in the nucleus, indicating that the localization of lncRNAs may also be regulated by m6A (Wu et al., 2019). ALKBH5 maintains the stability of the lncRNA GAS5-AS1, while YTHDF2/3 reduces its stability and accelerates lncRNA decay (Wang et al., 2019a; Ni et al., 2019). METTL16 introduces m6A into the U6 snRNA and regulates subsequent processing, thus regulating SAM homeostasis (Pendleton et al., 2017).

### Role of m6A in the Development of Multiple Organs

The dynamic m6A modification precisely regulates mRNA translation and degradation during early development. METTL3 mutations lead to early developmental stagnation, defects in the transition from mother to zygote and even embryonic lethality (Gu et al., 2021). Recent studies have focused on the role of m6A in the development of three main systems: the hematological system, nervous system and reproductive system. Further investigations are needed to determine whether m6A affects other systems. METTL3 deficiency affects hematopoietic development by significantly inhibiting the transition from endothelial cells to hematopoietic stem cells (HSCs) (Zhang et al., 2017b). In vascular endothelial cells, METTL3 knockout inhibits the function of hematopoietic stem/progenitor cells (HSPCs), while METTL3 knockout in HSPCs promotes differentiation (Vu et al., 2017; Lv et al., 2018).

The development of the nervous system depends on the specific expression of m6A modulators in different regions, cell subtypes and developmental stages of the brain. YTHDF2 deficiency in the embryonic neocortex impairs the self-renewal of neural stem/progenitor cells and special patterns of brain cell generation, leading to a failure of neural development (Yoon et al., 2017). METTL3 overexpression leads to structural disorders in both Purkinje and glial cells, and low METTL3 expression results in severe developmental defects in the cerebellum, indicating that a delicate m6A balance is essential for normal development (Ma et al., 2018; Wang et al., 2018). FTO knockout inhibits the proliferation and neuronal differentiation of adult neural stem cells and suppresses the expression of several crucial proteins involved in the brain-derived neurotrophic factor pathway, indicating its essential role in regulating adult neurogenesis (Li et al., 2017a).

Gametogenesis, a key step in reproductive system development, is also regulated by m6A modifications at the posttranscriptional level. YTHDC1/2 is required for spermatogenesis and oogenesis. YTHDC1 deficiency alters the length of the 3′ UTR by causing extensive alternative polyadenylation and impairs alternative splicing by inhibiting factors associated with premRNA 3′ end processing (Kasowitz et al., 2018). These events block oocytes at the primary follicle stage and eventually result in defective oogenesis. YTHDC2 knockout in germ cells leads to a failure to develop past the zygotene stage, thus resulting in male and female infertility (Hsu et al., 2017).

### Roles of m6A in Metabolism and Energy Homeostasis

m6A plays important roles in nutritional metabolism and energy balance, which are related to the pathogenesis of metabolism-related diseases, including type 2 diabetes and obesity (Gu et al., 2021). m6A activates glucose oxidation in rat adipocytes, suggesting that appropriate m6A levels may be essential for maintaining certain blood glucose concentrations (Souness et al., 1982). METTL3 knockout suppresses the expression of genes related to insulin secretion, thus inducing islet β-cell failure (Li et al., 2021b). METTL3 deficiency in mouse hepatocytes improves glucose tolerance and insulin sensitivity and decreases lipid accumulation (Li et al., 2020b). Acute deletion of METTL14 in β-cells reduces insulin secretion by increasing the activity of the IRE1a/sXBP-1 signaling pathway, finally leading to glucose intolerance (Men et al., 2019). Based on these results, METTL3/4 is essential for islet β-cell biology and maintaining the glucose balance. FTO is associated with carbohydrate and lipid metabolism and is involved in energy homeostasis. According to recent evidence, FTO participates in glucose metabolism through both m6A-dependent and nonm6A-dependent pathways (Wu et al., 2020). FTO also regulates adipogenesis by mediating the spicing of adipogenic regulatory factor RUNX1 translocation partner 1 (RUNX1T1), and AMPK positively regulates m6A levels in mRNAs to negatively regulate lipid accumulation in skeletal muscles (Zhao et al., 2014; Wu et al., 2017).

### Role of m6A in Immune Regulation

m6A has been reported to exert dual effects on regulating the immune response during both tumorigenesis and viral infection (Li et al., 2021a; Gu et al., 2021). m6A inhibits or promotes cancer progression by exerting dual-directional regulatory effects on apoptosis, autophagy, angiogenesis and the epithelial-mesenchymal transition (EMT). m6A modulators inhibit apoptosis by increasing oncogene expression levels and inhibiting tumor suppressor expression. In contrast, m6A modulators promote apoptosis by inhibiting the expression of oncogenes and promoting the expression of tumor suppressors (Li et al., 2021a). FTO silencing decreases the expression of light chain 3B (LC3B), a membrane marker of autophagy, but increases the expression levels of autophagy substrates by demethylating the UNC-51-like kinase 1 (ULK1) mRNA (Jin et al., 2018). Conversely, FTO promotes autophagy by directly demethylating autophagy-related (ATG) 5 and ATG7 (Wang et al., 2020b). In hepatocellular carcinoma, METTL3 knockdown induces the expression of some angiogenic biomarkers and increases the formation of tubes, indicating that METTL3 inhibits angiogenesis (Lin et al., 2020). IGF2BP3 recognizes m6A sites catalyzed by METTL3 and promotes angiogenesis by increasing the stability of HDGF transcripts (Wang et al., 2020c). Moreover, METTL3 overexpression decreases the expression of vimentin, β-catenin and N-cadherin and increases E-cadherin accumulation in renal cell carcinoma cells, thus promoting the EMT. Accordingly, METTL3 loss resulted in opposite alterations (Li et al., 2017b). Moreover, modulators alter the tumor microenvironment by regulating the expression of regulatory factors, such as lysosomal cathepsins and the TLR4 adaptor protein TIRAP, thus affecting immune escape and cancer immunotherapy (Li et al., 2021a). Based on the mechanism described above, m6A plays dual regulatory roles in tumorigenesis. However, the precise mechanism of the m6A modification in tumorigenesis remains to be fully elucidated.

Similarly, m6A promotes and inhibits immunity against viruses by modulating the lifecycles of viruses and immune responses of hosts (Gu et al., 2021). METTL3 increases replication efficiency by increasing the SUMOylation and ubiquitination of RNA polymerase 3D and is recruited to replication sites of viral RNAs(Hao et al., 2019). Moreover, writer and eraser knockdown promotes and inhibits the infection rate of the hepatitis C virus by increasing or decreasing the production of infectious viral particles, respectively (Gokhale et al., 2016). High METTL14 expression maintains the stability of latent Epstein-Barr virus transcripts (Lang et al., 2019). In hosts, METTL3 regulates innate immunity and adaptive immunity, including macrophages, dendritic cells (DCs), to exert a regulatory effect on viral infection (Gu et al., 2021).

## Role of m6A in Immune Cells

**FIGURE 2:**
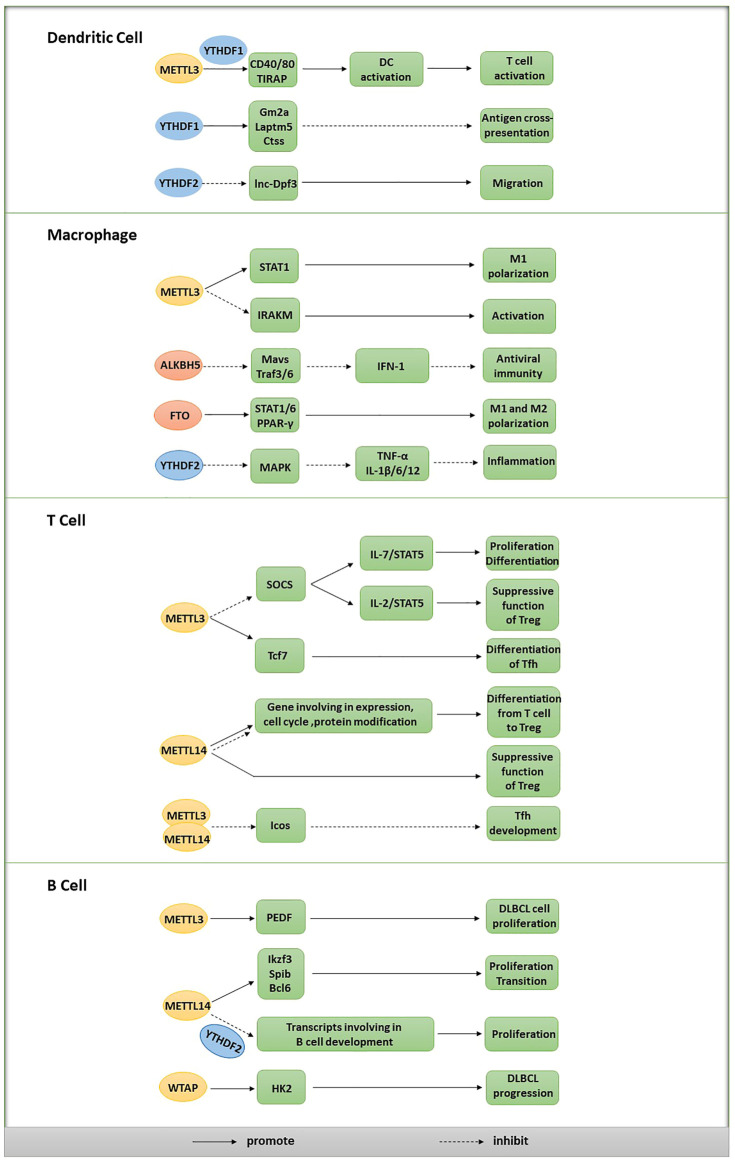
Effects of m6A on different types of immune cells.

The precursor hematopoietic stem cells located in the bone marrow are the original source of all immune cells in the blood, lymph and immune organs. Immune cells are classified as innate immune cells and adaptive immune cells according to their functions and patterns in the immune response. Innate immune cells primarily include DCs, macrophages, granulocytes and mast cells that respond rapidly to antigenic stimuli. Adaptive immune cells primarily include T lymphocytes (CD4^+^ T cells and CD8^+^ T cells) and B lymphocytes that initially exhibit a delayed response but are involved in the formation of immunological memory to respond strongly and rapidly to repeated stimulation with the same antigen. In addition, other types of immune cells, such as natural killer (NK) cells and NK-T cells, are components of both immune systems (McComb et al., 2019). Based on accumulating evidence, m6A is required for many processes in immune cells, including development, differentiation, activation, migration and polarization, thereby modulating the immune response (Figure 2).

### Role of m6A in DCs

Antigens are efficiently phagocytosed, processed and presented by DCs, leading to the activation of T cells and initiation of the immune response. METTL3, the catalytic subunit of the writer complex, is essential for the maturation and functional activation of DCs. METTL3 in DCs promotes T cell activation by catalyzing the formation of m6A in signaling molecule transcripts, including CD40, CD80 and TLR4 signaling adaptor (TIRAP). Then, these m6A-modified transcripts are recognized by YTHDF1 to increase their downstream translation, thus promoting DC activation and subsequent T cell responses (Wang et al., 2019b). On the other hand, YTHDF1 recognizes m6A-modified transcripts encoding lysosomal proteases and promotes their translation, thus limiting antigen cross-presentation by degrading protein antigens (Han et al., 2019). Therefore, YTHDF1 deficiency in DCs enhances antitumor immunity by promoting the cross-presentation of tumor antigens and cross-priming of CD8^+^ T cells, indicating that YTHDF1 is likely to become a promising antitumor target (Karthiya and Khandelia, 2020). CCR7 induces lnc-Dpf3, a lncRNA, to bind HIF-1α and suppress HIF-1-dependent transcription of glycolytic genes, thus suppressing CCR7-dependent DC migration. YTHDF2 recognizes m6A-modified lnc-Dpf3 and accelerates its degradation, which may exacerbate the inflammatory response and disrupt immune homeostasis by promoting DC migration (Liu et al., 2019a).

### Role of m6A in Macrophages

Macrophages perform various functions, including removing damaged, dead or dying cells and other debris, presenting antigens to cells and producing cytokines and other regulatory factors, similar to secretory cells, to modulate the immune response. Type I interferon (IFN-1) production is inhibited by DDX46, a DDX helicase, through its interaction with Mavs, Traf3 and Traf6 transcripts, which encode signaling molecules essential for IFN-1 production under viral stimulation. In infected macrophages, DDX46 recruits ALKBH5 through its DEAD helicase domain to catalyze the demethylation of these methylated transcripts, which leads to the retention of the unmodified transcripts in the nucleus, prevents their efficient translation, inhibits IFN-1 production and finally suppresses antiviral immunity (Zheng et al., 2017).

Macrophages can be polarized into the M1 and M2 phenotypes; M1 macrophages produce interferon γ (IFN-γ) to mediate proinflammatory activities, while M2 macrophages produce the cytokine interleukin-4 (IL-4) to mediate anti-inflammatory activities. The functional status of macrophages changes substantially with alterations between M1 and M2 polarization. The level of the METTL3 protein was reported to be specifically upregulated after M1 polarization of mouse macrophages. Furthermore, METTL3 directly methylates the mRNA encoding signal transducer and activator of transcription 1 (STAT1), an essential modulator of M1 polarization. Then, the stability of the methylated STAT1 mRNA is increased, and the STAT1 protein level is accordingly upregulated, thus driving M1 macrophage polarization. However, METTL3 deletion exerts opposing effects on macrophage polarization, decreasing M1 polarization but increasing M2 polarization and thus promoting an anti-inflammatory response (Liu et al., 2019b). In another study, FTO silencing induced the downregulation of STAT1 expression in M1-polarized macrophages and decreased the expression of STAT6 and PPAR-γ in M2-polarized macrophages. Specifically, FTO knockdown suppressed NF-κB signaling by downregulating the phosphorylation of related proteins and decreasing the mRNA stability of STAT1 and PPAR-γ through a mechanism dependent on the effect of YTHDF2, thereby preventing both M1 and M2 polarization of macrophages (Gu et al., 2020).

IRAKM is postulated to negatively regulate TLR4 signaling, which promotes macrophage activation. In another study, METTL3 deficiency led to impaired m6A formation in the IRAKM mRNA and slowed IRAKM degradation, ultimately inhibiting macrophage activation mediated by the suppression of TLR signaling (Tong et al., 2021). Upon lipopolysaccharide (LPS) stimulation, YTHDF2-deficient macrophages exhibited increased stability of the MAPK mRNA and its upregulated expression; the MAPK and NF-κB signaling pathways were subsequently activated to increase the expression levels of signaling molecules, including TNF-α, IL-1β, IL-6 and IL-12 (Liu et al., 2021).

### Role of m6A in T Cells

CD4^+^ T cells and CD8^+^ T cells are the primary classes of T cells. CD8^+^ T cells (cytotoxic T lymphocytes, CTLs) secrete cytotoxic granules and perforin into the immune synapse to induce apoptosis in target cells, including infected cells and tumor cells. CD4^+^ T cells (helper T cells, Th) differentiate into different phenotypes upon stimulation with different cytokines, and the differentiated cells contribute to cellular immunity or humoral immunity by secreting different cytokines. CD4^+^ T cells are activated by IL-12 and IFN-γ stimulation to differentiate into Th1 cells and secrete IFN-γ and lymphotoxin-alpha (LT-α) to induce inflammation and support cellular immunity, while CD4^+^ T cells are activated by IL-4 stimulation to differentiate into Th2 cells and secrete IL-4 to support humoral immunity and antibody production (McComb et al., 2019). In summary, Th1 cells induce a proinflammatory response, while Th2 cells induce an anti-inflammatory response. As another Th cell subtype, Th-17 cells produce IL-17 and promote inflammation and autoimmunity, while regulatory T cells (Tregs) promote immune tolerance, maintain immune homeostasis and suppress autoimmunity (Bettelli et al., 2007; Sakaguchi et al., 2008; Wan, 2010).

SOCS family proteins compete with IL-7 for binding to the IL-7 receptor, resulting in a failure to activate STAT5 and downstream signals that are important for the differentiation and proliferation of naïve T cells. METTL3 deletion reduces the m6A level in the SOCS mRNA and decreases SOCS mRNA degradation, leading to increased SOCS mRNA and protein levels. Accordingly, upregulated SOCS family activity inhibits STAT5 activation mediated by IL-7, eventually preventing the normal proliferation and differentiation of T cells. Moreover, m6A may contribute to the induction of SOCS mRNA degradation upon IL-7 stimulation to promote proliferation and differentiation by reprogramming naïve T cells (Li et al., 2017c). Another study showed that the depletion of METTL3 in Tregs increased the mRNA levels of SOCS family genes, suppressed the IL-2/STAT5 signaling pathway and impaired the suppressive function of Tregs (Tong et al., 2018b). Based on these results, the m6A RNA modification regulates the differentiation of naïve T cells and sustains the suppressive functions of Tregs by specifically targeting the same family of genes in different T cell subtypes. Additionally, in a mouse model of colitis, METTL14 deficiency in T cells increases inflammatory cell infiltration, increases cytokine release from Th1 and Th17 cells and prevents the differentiation of naïve T cells into Tregs (Lu et al., 2020).

T follicular helper (Tfh) cells are critical for the formation of germinal centers (GCs) and effective humoral immunity. METTL3 has been suggested to play a key role in modulating the expression of important Tfh signature genes, including Tcf7 and Icos, which are related to the development and differentiation of Tfh cells. The m6A modification was reported to increase the stability of Tcf7 transcripts to promote Tfh cell differentiation programs in a METTL3-dependent manner (Yao et al., 2021), and the METTL3/METTL14 complex was shown to catalyze m6A installation on the Icos mRNA and subsequently cause GAPDH protein-induced suppression of Icos expression, thereby inhibiting Tfh cell development (Zhu et al., 2019).

### Role of m6A in B Cells

B cells are the main cells mediating humoral immunity. B cells depend on their B cell receptors (BCRs) to recognize specific antigens and differentiate into plasma cells, which produce and secrete specific antibodies that bind to the target antigen (McComb et al., 2019).

According to recent studies, the m6A modification and its regulators may be involved in the early development and proliferation of B cells. For example, METTL14 deficiency dramatically decreases the m6A level in mRNAs and causes the aberrant expression of genes essential for B cell development, eventually inhibiting IL-7-induced pro-B cell proliferation and the transition from large pre-B cells to small pre-B cells (Zheng et al., 2020). Moreover, IL-7-induced pro-B cell proliferation depends on transcriptional suppression mediated by YTHDF2, while the failure to transition from large pre-B cells to small pre-B cells is independent of both YTHDF1 and YTHDF2 (Zheng et al., 2020). METTL3 is expressed at high levels in diffuse large B cell lymphoma (DLBCL) cell lines and patient tissues and increases the m6A levels in the pigment epithelium-derived factor (PEDF) transcript to promote DLBCL cell proliferation (Cheng et al., 2020). Similarly, WTAP upregulation induced by piRNA-30473 promotes DLBCL progression by increasing the m6A level in HK2 transcripts and the subsequent expression of HK2(Han et al., 2021).

## Role of m6A in Autoimmune Diseases

The role of immune cells in the pathogenesis of autoimmune diseases has been extensively studied, and these cells have proven to be involved in the development of autoimmune disorders. Interestingly, some immune cells, including macrophages, T cells and NK cells, exert dual effects: disease promotion and disease prevention (Table 2). This dual function may be attributed to differences in subsets of immune cells, the tissue microenvironment, and stages of autoimmune disease and to interactions between immune cells. Recent evidence shows that m6A may be involved in the development of autoimmune diseases. Moreover, some studies strongly indicate that m6A regulates the functions of immune cells, thereby affecting autoimmune diseases.

**TABLE 2:** Contribution of immune cells to the development of autoimmune diseases.

Cell type	Contribution	Autoimmune disease	References
T cell (except Treg)	Promotion	Rheumatoid arthritis	Cope et al. (2007); Han et al. (2015)
		Systemic lupus erythematosus	Han et al. (2015); Shao et al. (2020)
		Sjogren’s syndrome	Han et al. (2015)
		Inflammatory bowel disease	Han et al. (2015)
		Multiple sclerosis	Han et al. (2015)
		Type 1 diabetes	Shao et al. (2020)
Treg	Protection	Rheumatoid arthritis	Cope et al. (2007)
		Systemic lupus erythematosus	Ohl and Tenbrock, (2015)
		Sjogren’s syndrome	Alunno et al. (2015)
		Multiple sclerosis	Danikowski et al. (2017)
		Inflammatory bowel disease	Műzes et al. (2012)
		Type 1 diabetes	ElEssawy and Li, (2015)
B cell	Promotion	Rheumatoid arthritis	Rubin et al. (2019)
		Systemic lupus erythematosus	Rubin et al. (2019)
		Sjogren’s syndrome	Rubin et al. (2019)
		Type 1 diabetes	Rubin et al. (2019)
		Multiple sclerosis	Sabatino et al. (2019)
Dendritic cell	Promotion	Rheumatoid arthritis	Worbs et al. (2017)
		Systemic lupus erythematosus	Worbs et al. (2017)
		Psoriasis	Worbs et al. (2017)
		Type 1 diabetes	Diana et al. (2013)
		Multiple sclerosis	Mohammad et al. (2012)
Macrophage	Promotion	Rheumatoid arthritis	Funes et al. (2018); Shapouri-Moghaddam et al. (2018)
		Systemic lupus erythematosus	Funes et al. (2018)
		Autoimmune neuritis	Funes et al. (2018)
		Inflammatory bowel disease	Funes et al. (2018)
		Systemic sclerosis	Funes et al. (2018)
		Autoimmune hepatitis	Shapouri-Moghaddam et al. (2018)
		Crohn’s disease	Shapouri-Moghaddam et al. (2018)
		Multiple sclerosis	Shapouri-Moghaddam et al. (2018)
	Protection	Systemic lupus erythematosus	Funes et al. (2018)
		Autoimmune neuritis	Funes et al. (2018)
		Inflammatory bowel disease	Funes et al. (2018)
NK cell	Promotion	Multiple sclerosis	Fogel et al. (2013)
		Rheumatoid arthritis	Palm et al. (2015)
		Psoriasis	Ottaviani et al. (2006)
		Primary biliary cirrhosis	Shimoda et al. (2011)
		Type 1 diabetes	Dotta et al. (2008)
	Protection	Multiple sclerosis	Shi et al. (2011)
		Type 1 diabetes	Dotta et al. (2008)

### Rheumatoid Arthritis

High-throughput m6A sequencing revealed a potential relationship between RNA methylation and rheumatoid arthritis (RA)-related genes, suggesting that m6A may contribute to the initiation and development of RA. Indeed, the global m6A content in peripheral blood is significantly increased in patients with RA compared to healthy people. Quantitative real-time polymerase chain reaction showed that the mRNA expression levels of ALKBH5, FTO and YTHDF2 were decreased in peripheral blood mononuclear cells (PBMCs) isolated from patients with RA. However, ALKBH5 expression is upregulated in patients with RA after treatment with the appropriate drug therapy. In addition, associations were identified between FTO mRNA expression and some indicative markers of RA activity, including the IgG level, C3 level, disease activity score 28 (DAS28) score and lymphocyte-to-monocyte ratio (LMR). Moreover, associations were observed between YTHDF2 mRNA expression and the red blood cell (RBC) count, lymphocyte percentage (L%), neutrophil percentage (N%), neutrophil-to-lymphocyte ratio (NLR), and LMR. In summary, m6A and regulators such as ALKBH5, FTO and YTHDF2 may be promising candidates for assessing the risk and progression of RA (Luo et al., 2020a).

METTL3 expression is significantly upregulated in patients with RA. In addition, positive correlations were found between METTL3 expression and biochemical indexes, including the C-reactive protein (CRP) level and erythrocyte sedimentation rate (ESR), which suggested alterations in RA disease activity. LPS stimulation of macrophages increases the expression and biological effects of METTL3. Moreover, METTL3 overexpression significantly inhibits the LPS-induced inflammatory response in macrophages through the NF-κB pathway (Wang et al., 2019). However, METTL3 may promote the activation of fibroblast-like synoviocytes (FLSs) and the inflammatory response through the NF-κB pathway, thus accelerating the initiation and progression of RA (Shi et al., 2021). Therefore, the precise role of METTL3 in the pathogenesis of RA remains to be further investigated.

### Systemic Lupus Erythematosus

A comprehensive review first proposed that a link between the m6A modification and systemic lupus erythematosus (SLE) is reasonable based on the observation that m6A effectively regulates gene expression and the immune system (Li et al., 2018). Other scientists then observed downregulated mRNA expression of m6A regulators, including METTL3, METTL14, WTAP, FTO, ALKBH5 and YTHDF2, in patients with SLE (Luo et al., 2020b; Luo et al., 2020c). These decreases correlated with the index used to predict SLE disease activity. Specifically, levels of the METTL14 and YTHDF2 mRNAs in patients with SLE were associated with CRP and C3 levels, while the ALKBH5 mRNA levels of in patients with SLE were associated with C3, CRP and autoantibody levels and skin manifestations. In addition, positive correlations were observed among mRNA levels of three different regulators in PBMCs from patients with SLE (Luo et al., 2020b; Luo et al., 2020c). In addition, logistic regression and multivariate logistic regression analyses revealed that downregulated expression of the YTHDF2 or ALKBH5 mRNA may be associated with an increased risk of developing SLE (Luo et al., 2020b; Luo et al., 2020c). These findings indicate that the m6A regulators ALKBH5 and YTHDF2 are likely to be involved in the pathogenesis of SLE and are expected to be effective biomarkers to assess the SLE risk and disease activity (Luo et al., 2020c).

### Multiple Sclerosis

In a comprehensive analysis of DNA methylation and gene expression data, Mo et al. identified rs923829 in METTL21B and rs2288481 in the DKKL1 gene as strongly correlated with multiple sclerosis (MS). An analysis of the HaploReg database showed that these two m6A-related SNPs regulate the expression of the METTL21B and DKKL1 genes. Then, the researchers selected PBMCs from a small group of Chinese participants to validate the association between rs923829 and METTL21B expression and between rs2288481 and DKKL1 expression. Importantly, rs923829 is strongly associated with METTL21B expression, while a significant statistical association is not observed between rs2288481 and DKKL1 expression. This group proved that these m6A-related SNPs may be related to the pathogenesis of MS(Mo et al., 2019).

Experimental autoimmune encephalomyelitis (EAE) is internationally recognized as an animal model for studying MS. More recently, specific ablation of ALKBH5 in T cells conferred protection against EAE. Mechanistically, the m6A eraser ALKBH5 decreased the m6A levels in the CXCL2 and IFN-γ mRNAs and subsequently increased their transcript stability and protein expression, thereby enhancing CD4^+^ T cell-mediated responses and inflammatory cell infiltration in the central nervous system to induce neuroinflammation (Zhou et al., 2021). This study was the first to prove a direct link between autoimmunity and m6A-mediated functions of immune cells.

### Psoriasis

Transcriptome-wide m6A profiling revealed that transcripts from psoriatic skin had the fewest m6A peaks and lowest m6A peak density compared with transcripts from uninvolved psoriatic skin and healthy skin. Bioinformatics pathway analyses indicated that transcripts that were hypermethylated in psoriatic skin were primarily correlated with inducing various responses, including immune responses, cytokine production and olfactory signal transduction, while transcripts that were hypomethylated in psoriatic skin were strongly related to the Wnt signaling pathway and development-related processes. Transcripts with lower expression levels were preferentially modified with m6A. Moreover, gene expression was upregulated in psoriatic skin, accompanied by increased m6A levels, indicating that alterations in m6A methylation affect the gene expression pattern (Wang and Jin, 2020).

### Other Autoimmune Diseases

T cell-specific METTL14 deficiency prevents the differentiation of naïve T cells into Tregs, leading to an imbalance in Th17 cells and Tregs, and thereby inducing spontaneous colitis. Considering that dysregulation of the balance between Th17 cells and Tregs is strongly associated with the initiation of inflammatory bowel disease (IBD), a reasonable assumption is that METTL14 may be involved in IBD development (Lu et al., 2020). Another study showed that CD4^+^ T cells induce autoimmune colitis, an ability that might be controlled by ALKBH5. Therefore, ALKBH5-deficient naïve CD4^+^ T cells failed to migrate into colon tissue, and their ability to promote colitis was reduced (Zhou et al., 2021).

Variants in IGF2BP2 were shown to decrease glucose-stimulated insulin secretion in the first phase of diabetes development. Additionally, IGF2BP2 was found to be downregulated and linked to diabetic nephropathy in male patients with type 1 diabetes (T1D), as well as impaired glucose tolerance in patients with type 2 diabetes (T2D) (Wang et al., 2021b). Correlations were observed between polymorphisms in the ALKBH5 gene, including rs9913266 and rs12936694, and the development of autoimmune thyroid disease (AITD). Therefore, ALKBH5 might be a candidate susceptibility gene for AITD (Song et al., 2021).

## Conclusions and Future Perspectives

Over several decades, studies of m6A modifications have resulted in substantial progress in epitranscriptomics. Convincing evidence suggests that reversible m6A modification may be involved in regulating many processes in immune cells, including development, differentiation, activation, and migration. Based on these results, m6A may participate in the pathogenesis of immune-related diseases, including cancers, viral infections, and inflammatory and autoimmune diseases. Currently, the relationships between m6A and cancer and viral infection have received extensive research attention. However, studies examining the essential role of the m6A modification in the pathogenesis of autoimmune diseases are lacking, although existing evidence strongly indicates that m6A may be involved in the development of autoimmune diseases. Direct studies assessing the mechanisms by which and to what extent m6A contributes to autoimmune diseases are urgently needed. In addition to the pathogenesis of autoimmune diseases, we should focus more on achieving the transition from mechanistic research to clinical applications, including diagnosis and treatment. Therefore, the following research gaps still remain to be filled to provide new opportunities for the treatment of immune-related diseases, including autoimmune diseases.

1. Innovative and more advanced technology. Various limitations in current technology exist, including low precision, poor calculation methods, high complexity, low repetition. Scientists must develop more convenient and accurate sequencing and imaging technology for the rapid and quantitative detection of the m6A modification, perform functional analysis and understand the dynamic mechanisms of modified RNAs.

2. Precise regulatory mechanisms among m6A modulators. Although numerous findings related to the function of m6A modulators have been reported, many knowledge gaps remain to be filled. The dynamic expression pattern of modulators makes functional identification more complicated. We should understand the mechanisms mediating the spatiotemporal specificity of m6A, how the function of regulatory proteins is regulated in different cell types, how to precisely regulate different target RNAs, how mediators regulate interactions with other regulatory proteins to perform their respective functions or exert their comprehensive effects, and how their unbalanced deposition leads to pathological processes.

3. Complicated network between m6A and other regulatory factors. m6A modification and other epigenetic regulatory mechanisms, including chromatin state interaction and histone modification, are emerging as a new area in the epitranscriptomic field. In addition, m6A exerts a decisive effect on the fate of noncoding RNAs, including microRNAs, lncRNAs and circRNAs. Their interaction will prompt more studies to obtain an in-depth understanding of m6A. Moreover, the network between m6A and other RNA modifications, including 5-methylcytosine (m5C) and pseudouridine (Ψ), should be further explored.

4. Promizing but difficult clinical applications. m6A provides novel insights into the diagnosis, treatment and prognosis of diseases, especially autoimmune diseases. However, the use of modulators as therapeutic agents remains an important challenge. First, in the present review, m6A exerts dual effects on immune-related diseases, indicating a lack of consistent and consolidated evidence. Second, its safety has not been guaranteed. Finally, few small-molecule stimulants or inhibitors targeting m6A are available. Therefore, efforts are urgently needed to screen molecular drugs targeting m6A. An understanding of the mechanisms by which RNA modifications are introduced, removed or read will reveal the underlying pathogenesis and provide therapeutic targets for autoimmune diseases in the future.
